# Myths and reality of HPbI_3_ in halide perovskite solar cells

**DOI:** 10.1038/s41467-018-07204-y

**Published:** 2018-11-14

**Authors:** Weijun Ke, Ioannis Spanopoulos, Constantinos C. Stoumpos, Mercouri G. Kanatzidis

**Affiliations:** Department of Chemistry, Northwestern University, Evanston, IL 60208 USA

## Abstract

All-inorganic perovskites have a special place in halide perovskite family because of their potential for better stability. However, the representative cesium lead iodide (CsPbI_3_) is metastable and spontaneously converts to the non-perovskite structure at room temperature. Here, we demonstrate that what appears to be all-inorganic CsPbI_3_ stabilized in its perovskite form using the purported intermediate known as hydrogen lead iodide (HPbI_3_) is, in fact, the hybrid perovskite cesium dimethylammonium lead iodide (Cs_1−*x*_DMA_*x*_PbI_3_, *x* = 0.2 to 0.5). Thus, many of the reported all-inorganic perovskites are actually still hybrid organic-inorganic perovskites, as strongly evidenced by a wide battery of experimental techniques presented here. Solar cells based on the representative composition Cs_0.7_DMA_0.3_PbI_3_ can achieve an average power conversion efficiency of 9.27 ± 1.28% (max 12.62%). These results provide an alternative angle to look at previous results pertaining all-inorganic CsPbI_3_ while the DMA cation is now revealed as an alternative A site cation.

## Abstract (web summary)

Hydriodic acid or hydrogen lead iodide is widely used to stabilize all-inorganic perovskite cesium lead iodide to make high performing solar cells. Here Ke et al. reveal the real composition of the perovskites, where dimethylammonium partially take place of cesium cation at the A-site.

## Introduction

Halide perovskites have unique properties such as tunable band gaps, long diffusion lengths, high optical absorption coefficients, and low exciton binding energies^1–8^. Halide perovskite solar cells have achieved a record power conversion efficiency (PCE) of 22.7%^9^, which are a great candidate for the next generation of photovoltaics. High-performance perovskite solar cells typically employ organic-inorganic hybrid Pb-based perovskites as light absorbers such as methylammonium lead iodide (MAPbI_3_), formamidinium lead iodide (FAPbI_3_), and their mixtures, due to their excellent optical and electrical properties^10–16^. However, the intrinsic thermal and light instabilities of organic cations of MA and FA present serious hurdles in the further development and commercialization of long-term operating devices^17^. The all-inorganic perovskite analog cesium lead iodide (CsPbI_3_) could present an important way forward, since it possesses a relatively wider band gap with large band widths and especially a significantly better thermal stability compared with the organic cations-based perovskites^17–20^. However, the black CsPbI_3_ perovskite is metastable and it spontaneously converts to the undesired yellow polytype (δ-phase, NH_4_CdCl_3_ structure-type) at room temperature^5,18,21,22^. Yellow δ-CsPbI_3_ has a wide band gap of *E*_g_ = 2.82 eV and has poor transport properties^18^. By contrast, black CsPbI_3_ has a narrow band gap of around 1.7 eV and it is suitable for solar cell applications^21,23^. Therefore, much effort has been devoted to improving the phase-stability of the black-phase CsPbI_3_ perovskites so that highly efficient all-inorganic solar cells can be made^18,24–35^. In one approach, researchers used pre-synthesized CsPbI_3_ nanocrystals to make solar cells. For example, Protesescu et al. demonstrated that the black CsPbI_3_ perovskite (γ-phase) could be stabilized as nanocrystals^36^, due to the large contribution of surface energy. Subsequent work by Luther et al. demonstrated that colloidal CsPbI_3_ quantum dots synthesized by a hot-injection method can be phase-stable for several months in ambient air and the solar cells achieved a PCE as high as 13.43%^27,28^. In another approach, You et al. used dimethylformamide (DMF)/dimethyl sulfoxide (DMSO) solvent followed by high-temperature annealing process to obtain the black α-phase CsPbI_3_^33^. These solar cells yielded a record PCE of 15.7% and showed a good light-soaking stability under the protection of N_2_-filled glovebox. Much more commonly, the use of hydriodic acid (HI)^18,26^ or the so-called hydrogen lead iodide (HPbI_3_) precursor^31,37^, produced by HI treatment, are the most used approaches to stabilize the black α-CsPbI_3_. The use of HPbI_3_ was first reported as an intermediate in the synthesis of FAPbI_3_ solar cells^38^, and was prepared by mixing PbI_2_ and HI in DMF. Eperon et al. subsequently reported that HI can be used directly as an additive in DMF solutions of CsPbI_3_ to stabilize the black α-CsPbI_3_ films at room temperature^18^. The planar device made from this black CsPbI_3_ film achieved a PCE of 2.9%. Recently, Zhang et al. reported the formation of black CsPbI_3_ by a low-temperature deposition using the pre-synthesized PbI_2_·*x*HI, which was also made from DMF, PbI_2_, and HI^31^. Combined with the use of larger ethylenediammonium (*en*) cation, the champion α-CsPbI_3_ solar cell yielded a PCE of 11.8% with enhanced stability and good reproducibility^31^. More recently, Jiang et al. also used HPbX_3_ (*X* = I, Br) as the precursor together with phenylethylammonium (PEA) cation to fabricate low-dimensional α-CsPbI_3_ and the solar cells achieved a high PCE of 12.4%^37^. Despite the apparent success of the HPbI_3_ methodology, the mechanism of the stabilization of the black CsPbI_3_ by HI and HPbI_3_ remains unclear. The claim alone that HPbI_3_ even exists is extraordinary and there is no structural or spectroscopic validation for the existence of this compound. From the chemistry point of view, HPbI_3_ would be a solid acid with HI fragment in it and intuitively is unlikely to exist. This is because the HI molecule itself is not known to engage in binding to metals and it is likely that cannot form a stable structure^39^ and will readily dissociate to PbI_2_ and HI. Thus, the claim that HPbI_3_ is a real compound deserves closer attention and we set out to investigate it in the context of the present manuscript.

Here, we report that HPbI_3_ does not exist and instead what was believed to be it is in fact a compound of DMAPbI_3_ (DMA = dimethylammonium, (CH_3_)_2_NH_2_^+^), where DMA is a decomposition product of the acidic hydrolysis of DMF catalyzed by HI. We demonstrate that the black CsPbI_3_ films deposited from DMF containing HI/HPbI_3_ and reported to be all-inorganic black phase of CsPbI_3_ are not. Instead, they are the mixed-cation perovskite phase of cesium dimethylammonium lead iodide (Cs_1-*x*_DMA_*x*_PbI_3_, *x* = 0.2 to 0.5). The α-Cs_1-*x*_DMA_*x*_PbI_3_ perovskite films have similar characteristics with the black γ-CsPbI_3_ films arising from HI addition, but they exhibit better charge transport due to the superior band structure characteristics (larger bandwidth, smaller band gap) of the cubic phase. Our best-performing solar cell based on α-Cs_0.7_DMA_0.3_PbI_3_ perovskite absorber achieves a remarkable PCE of 12.62%. Our results reveal the existence of α-Cs_0.7_DMA_0.3_PbI_3_ a black 3D perovskite which is a great absorber for the fabrication of high efficiency solar cells.

## Results

### Phase diversity of CsPbI_3_

**Fig. 1:**
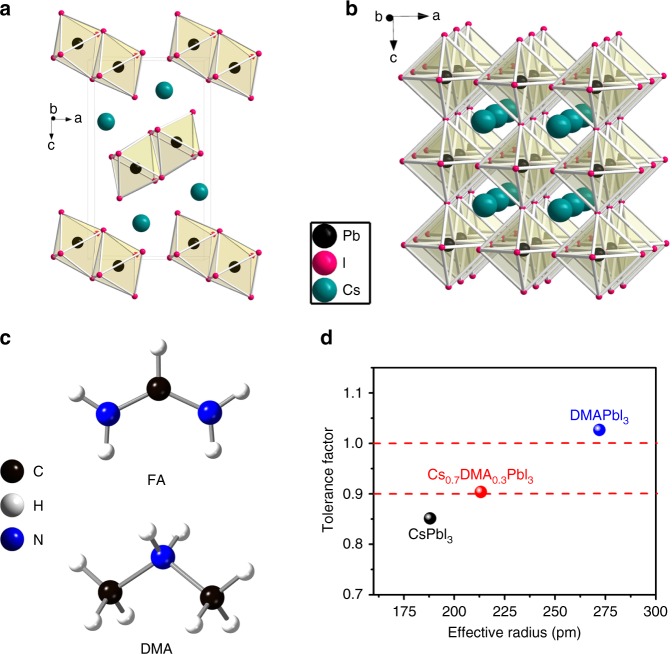
Crystal and molecular structures. **a** Crystal structure of CsPbI_3_ with non-perovskite yellow orthorhombic (δ) phase. **b** Crystal structure of CsPbI_3_ with perovskite black cubic (α) phase. **c** Molecular structures of FA (top) and DMA (bottom) cations. **d** Tolerance factors of CsPbI_3_, DMAPbI_3_, and Cs_0.7_DMA_0.3_PbI_3_

The structure of halide perovskites which have a general formula of AMX_3_ can be only stabilized by limited cations, according to the rule of tolerance factor, *t*^39,40^. Where A, M, and X represent a nonbonding univalent cation, an octahedrally coordinated bivalent metal ion, and a monoanionic halide ion, respectively^5,41^. A cubic structure of perovskite materials typically has a suitable *t* value ranged from 0.9 to 1.0, which is defined by the equation of *t* = (*r*_A_ + *r*_X_)/√2(*r*_M_ + *r*_X_). *r*_A_, *r*_M_, and *r*_X_ represent the ionic sizes of A, B, and X, respectively^42^. Different A cations have different ionic sizes, only cubic MAPbI_3_ could be stable at room temperature. Both FAPbI_3_ and CsPbI_3_ normally adopt the yellow phase at room temperature, due to the too large and too small size of A cations, respectively^5,42^. Specifically, CsPbI_3_ can adopt two structures:^21^ the NH_4_CdCl_3_-type yellow phase (δ-phase) stable at room temperature (Fig. 1a) and the black cubic CaTiO_3_-type (α-phase) structure stable above 300 °C (Fig. 1b). The perovskite structure can be kinetically stabilized at room temperature, where it adopts the black orthorhombic γ-phase (bandgap of around 1.73 eV) but it converts within hours to the δ-phase^5,42,43^. Thus, CsPbI_3_ is typically stabilized by adjusting the *t* via formation of solid solutions such as partial replacement of I with smaller Br anions to form CsPbI_3−*x*_Br_*x*_ compositions, which exhibit a wider band gap^44–54^, or by substituting the A-site with larger organic cations such as FA to form Cs_1−*x*_FA_*x*_PbI_3_ solid solutions^55^. DMA (2.72 Å) is very similar in shape to FA (2.53 Å) but has a slightly larger effective radius owing to the conjugated bonding in the latter (Fig. 1c)^56,57^. Because of this, pure DMAPbI_3_, which has a large *t* = 1.026 and is not able to form the perovskite structure, unlike FAPbI_3_. Pure DMAPbI_3_, which is identical to what previously reported as HPbI_3_, by inspection of the reported and simulated powder diffraction patterns (PXRD), has a 1D structure assembled by face-sharing [PbI_6_]^4−^ octahedra^58^, and it is isostructural with yellow FAPbI_3_^5^. Unlike FAPbI_3_, DMAPbI_3_ cannot be isolated as a black perovskite due to the too large size of DMA which leads to a prohibitively large *t*, even at elevated temperatures. On the other hand, the effective radius of Cs cation is only 1.88 Å and pure CsPbI_3_ has a low tolerance factor of *t* *=* 0.851 which is below the ideal range of cubic structure (Fig. 1d)^17,19^. However, mixing Cs with DMA, i.e., Cs_1-*x*_DMA_*x*_PbI_3_, can on average bring the effective tolerance factor within the desirable region, thus enabling the stabilization of a black perovskite phase, namely Cs_1−*x*_DMA_*x*_PbI_3_ (*x* = 0.2 to 0.5), having a *t* *=* 0.904 (*x* = 0.3) that allows it to adopt the cubic α-phase (Fig. 1d), similarly to Cs_1−*x*_FA_*x*_PbI_3_.

### Cs_1-*x*_DMA_*x*_PbI_3_ film properties

**Fig. 2:**
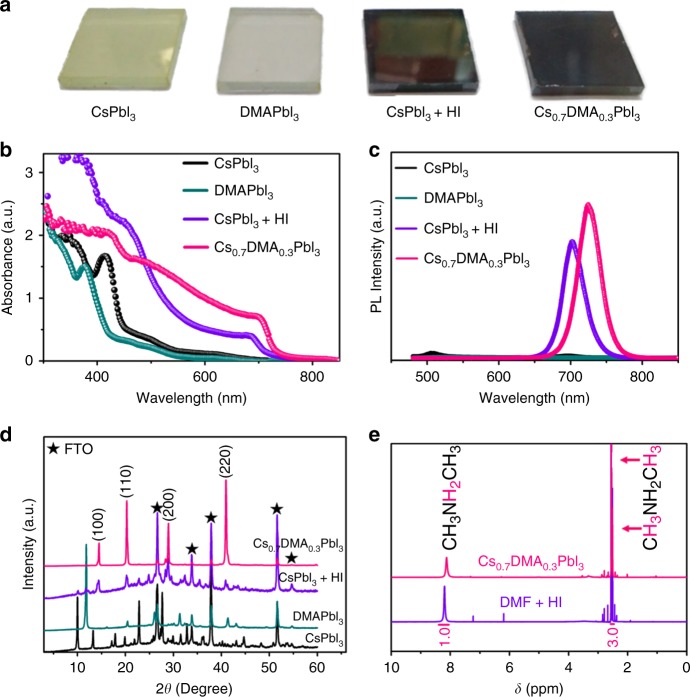
Film properties and component studies. **a** Photographs, **b** UV-vis absorption spectra, **c** PL spectra, and **d** XRD patterns of a pure CsPbI_3_ film, a pure DMAPbI_3_ film, a HI-treated CsPbI_3_ film, and a Cs_0.7_DMA_0.3_PbI_3_ film. **e** NMR spectra of the powder obtained from scratching away Cs_0.7_DMA_0.3_PbI_3_ films and DMAI polycrystalline powder synthesized from DMF and HI, which were dissolved in DMSO-d_6_

This has indeed turned out to be the case in our thin films, where the nominal Cs_0.7_DMA_0.3_PbI_3_ composition is able to adopt the perovskite phase. As shown in Fig. 2a, the Cs_0.7_DMA_0.3_PbI_3_ film without HI addition shows a black color, indicting the successful fabrication of black-phase perovskite structure with lattice constant *a* = 6.272 Å and *Pm*-3*m* space group. On the contrary, the pristine CsPbI_3_ and DMAPbI_3_ films deposited on FTO/PEDOT substrates are yellow and pale-yellow color (Fig. 2a), respectively, because they are non-perovskite NH_4_CdCl_3_ structure-type^56^. For comparison, we also fabricated CsPbI_3_ film with HI addition (see details in Methods). Similar to the previously reported results^18,25,26,59^, the CsPbI_3_ film prepared with HI addition also shows a black color and a shiny surface (Fig. 2a), indicative of the formation of the similar black-phase perovskite structure. The color of the films is in good agreement with the band gaps of the respective compounds as determined by optical absorption measurements. Fig. 2b shows that the Cs_0.7_DMA_0.3_PbI_3_ film has an absorbance onset at around 730 nm, indicating a band gap of 1.7 eV. The CsPbI_3_ film with HI addition shows a similar absorption but slightly blue-shifted from 730 nm to 710 nm. As expected, the pure CsPbI_3_ and DMAPbI_3_ films only show an absorption at around 440 nm and 390 nm, respectively, due to the wide band gaps of these yellow phases^18^. Photoluminescence (PL) measurements reveal the same trend. As shown in Fig. 2c, the Cs_0.7_DMA_0.3_PbI_3_ film has a PL emission at around 724 nm. The black CsPbI_3_ film with HI addition shows a blue-shift PL peak at around 704 nm, consistent with the trend of UV-vis absorption spectra. Both CsPbI_3_ and DMAPbI_3_ films only show negligible PL emission, due to the indirect nature of the band gaps.

X-ray diffraction (XRD) patterns on the thin-films confirm the structure description discussed above. As shown in Fig. 2d, the main peaks of the neat yellow CsPbI_3_ film locates at 9.9°, 13.2°, 22.8° etc. are assigned to the orthorhombic (*Pnma*) yellow δ-phase (Supplementary Fig. 1)^18^. The XRD pattern of the DMAPbI_3_ film only shows two main reflections at 2*θ* = 11.8° and 20.4° (Fig. 2d), due to the preferred orientation, corresponding to the hexagonal (*P*6_3_/*mmc*) yellow phase (Supplementary Fig. 2). The Cs_0.7_DMA_0.3_PbI_3_ film shows peaks at 2*θ* = 14.4°, 20.2°, 28.9°, and 40.9° etc., which can be indexed to the (100), (110), (200), and (220) reflections of cubic CsPbI_3_, showing some orientation of the perovskite in both (100) and (110) directions of the cubic (*Pm*-3*m*) α-phase (Supplementary Fig. 3)^18,30^. While the CsPbI_3_ film with HI additive exhibits similar peaks, it has no preferred orientation showing reflections that correspond to the black orthorhombic (*Pbnm*) perovskite γ-phase (Supplementary Figs. 4–5). In addition, the CsPbI_3_ film with HI also reveals a weak reflection at 2*θ* = 9.9°, which corresponds to the yellow δ-phase, indicating that the conversion to the black phase is incomplete.

To further prove that DMA cation is actually present in the black CsPbI_3_ films, we measured the proton nuclear magnetic resonance (^1^H NMR) spectrum of the powder obtained from scratching away the Cs_0.7_DMA_0.3_PbI_3_ films and dissolving it in dimethyl sulfoxide-d_6_ (DMSO-d_6_) (Fig. 2e). The signals of –NH_2_^+^– (singlet) and –CH_3_ (doublet) protons are located at *δ* = 8.17 ppm and *δ* = 2.55 ppm, respectively. The ratio integrated from the signals of –NH_2_^+^– and –CH_3_ is 1:3, indicating that DMA is protonated. The NMR spectrum of the pure DMAI powder confirms the peak position (Supplementary Fig. 6), thus demonstrating DMA is incorporated in the CsPbI_3_ structure as would be expected by the Cs_0.7_DMA_0.3_PbI_3_ composition. Remarkably, the NMR spectrum of the DMSO-d_6_ solution of the powder obtained from scratching away the CsPbI_3_ film with HI addition also shows the same two peaks in the 1:3 for –NH_2_^+^– and –CH_3_ ratio (Supplementary Fig. 7).

### The chemical mechanism and reality of so-called HPbI_3_

Thus, it transpires from the NMR results that the DMA cation is produced via a side reaction (of DMF with HI) and then gets incorporated in the CsPbI_3_ films. Since only CsI and PbI_2_ were dissolved in the DMF solvent, the only possible source of DMA in the precursor solution is DMF itself, which is known to slowly hydrolyze to DMA and formic acid (HCOOH)^60–65^. Addition of HI significantly accelerates the hydrolysis according to Eq. 1, heavily shifting the equilibrium towards the dissociation products, thus generating significant amounts of DMA in the mixture^66^.

1 $${\mathrm{HCON}}({\mathrm{CH}}_3)_2 + {\mathrm{HI}} + {\mathrm{H}}_2{\mathrm{O}} \to ({\mathrm{CH}}_3)_2{\mathrm{NH}}_2{\mathrm{I}}({\mathrm{DMAI}}) + {\mathrm{HCOOH}}$$

In order to further demonstrate this reaction, we synthesized the DMAI from the reaction of DMF and HI directly (see Methods). As Fig. 2e illustrate, DMAI can indeed be readily formed by the reaction between DMF and HI, with ^1^H-NMR spectra and XRD patterns confirming its identity in comparison with the commercial DMAI compound (Supplementary Figs. 6 and 8). Note that the two peaks at *δ* = 7.22 ppm and *δ* = 6.18 ppm shown in Fig. 2e are assigned to the –PH_2_– protons of hypophosphorous acid (H_3_PO_2_) which presents as a stabilizer in concentrated HI aqueous solutions. Therefore, this is the reason why ^1^H-NMR spectra of Cs_0.7_DMA_0.3_PbI_3_, CsPbI_3_ with HI, DMAI, and DMF + HI samples are identical. Most importantly, it explains why the UV-vis absorption, PL, XRD, and ^1^H-NMR results between the intentionally made Cs_0.7_DMA_0.3_PbI_3_ film and the DMF derived CsPbI_3_ with HI addition films are so similar. Note that the red shift (around 20 nm) of PL and UV-vis spectra of Cs_0.7_DMA_0.3_PbI_3_ with respect to the CsPbI_3_ sample with HI is because the former (which contains significant amounts of DMA) adopts the cubic α-CsPbI_3_ phase, whereas the latter (containing only small amounts of DMA) stabilizes the orthorhombic γ-phase (as would be expected by the effective tolerance factor discussed above).

The hydrolysis of DMF in the presence of acid such as HI as a film fabrication tool has great implications in the fabrication of perovskite films and solar cells. Since HI addition has been reported to alter the phase transition temperature and improve the film morphology of MAPbI_3_^67^, and also was identified as the critical reagent for stabilizing the perovskite phase of CsPbI_3_, the nature and exact composition of these crystalline films will need to be re-examined^18,25,26,59^. In addition, HPbI_3_, obtained from the reaction of HI, DMF, and PbI_2_, has been widely used for making stable FAPbI_3_^38^, CsPbI_3_^31,37,53^, and MAPbI_3_ solar cells^68^, which actually does not exist and should be reformulated to DMAPbI_3_. It is confirmed by the XRD patterns of our DMAPbI_3_ film (Fig. 2d and Supplementary Fig. 2) and the reported HPbI_3_^38^, showing the exact same reflections. Given that the use of HPbI_3_ in a serendipitous manner, these systems also need to be revisited and studied carefully under the understanding of the DMF hydrolysis mechanism. It is important to re-evaluate these systems counting the time as a synthetic parameter since the preparation will significantly vary over time as different amounts of DMA are produced at different times of the reactions and therefore the composition of the films will vary accordingly.

In a previous report, we demonstrated that HI addition can alter the MAPbI_3_ phase transition temperature^67^. A room temperature phase transition from tetragonal to cubic was observed for MAPbI_3_ films treated with high HX (X = Cl, Br, I) concentrations. In our original hypothesis, we and others speculated that the reduction of the crystallite size induced by the acid were responsible for this phase transition^18,67^. HI can improve the perovskite film morphology and eliminate the hysteresis of MAPbI_3_ solar cells^69,70^. However, in light of the results reported here, we can now revise our original concept and ascribe the compositional phase transition to the presence of DMA, induced by the presence of acid. Note that the concept of the role of acid expands beyond the HX acids, as other acids such as H_3_PO_2_^71^ appear to have a similar effect. Therefore, based on the above results, all those compositionally-induced phase transitions may be attributed to the introduction of the in-situ forming DMA cation in the perovskite lattice and it applies equally well in the MAPbI_3_ and CsPbI_3_ systems. Note that FAPbI_3_ is excluded since FA is already too large to stabilize the perovskite structure.

**Fig. 3:**
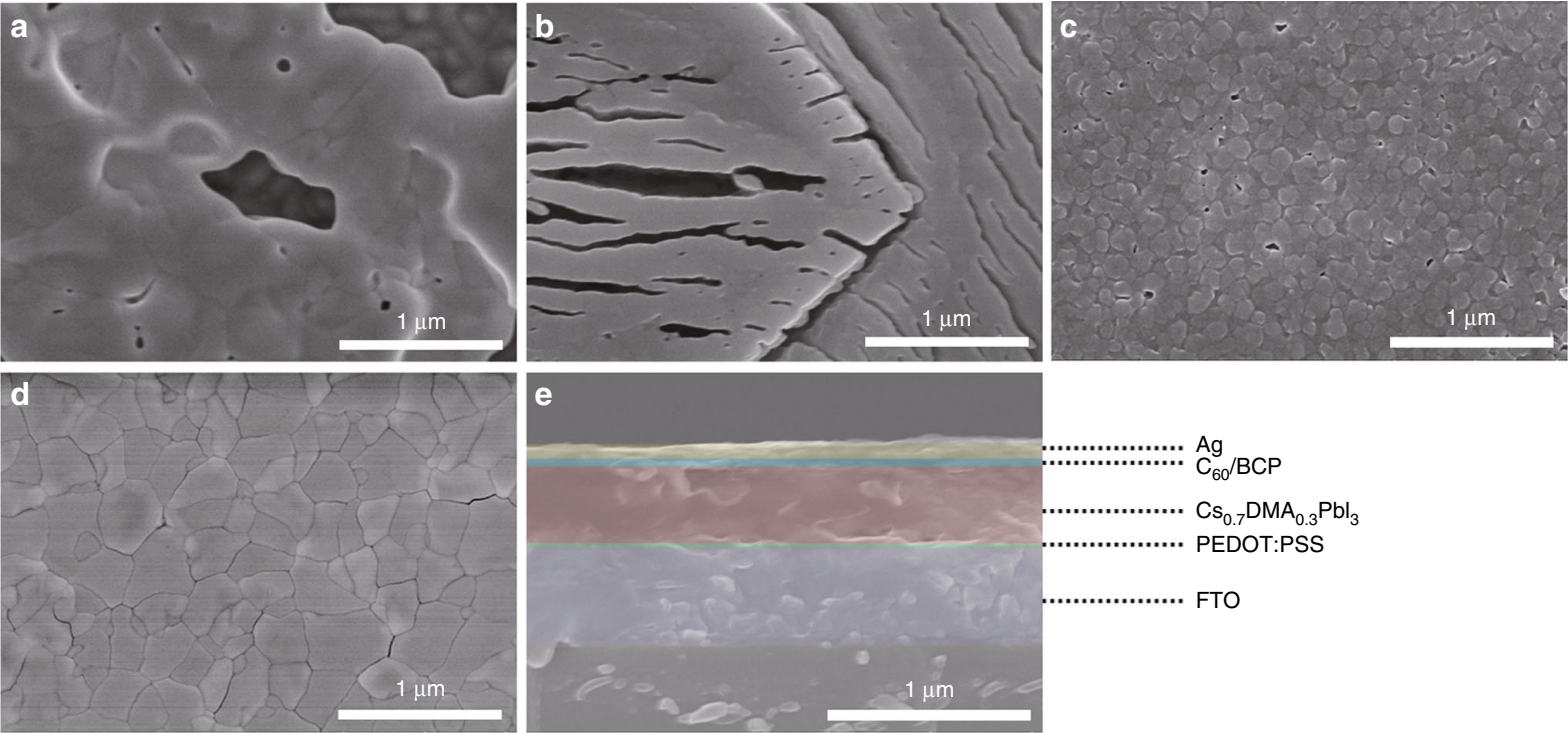
Film and device morphology. Top view SEM images of **a** a CsPbI_3_, **b** a DMAPbI_3_, **c** a HI-treated CsPbI_3_, and **d** a Cs_0.7_DMA_0.3_PbI_3_ film deposited on FTO/PEDOT substrates. **e** Cross-sectional SEM image of a completed device using a PEDOT:PSS hole transporting layer, a Cs_0.7_DMA_0.3_PbI_3_ absorber, a C_60_/BCP electron transporting layer, and a Ag metal electrode

Now that we have established that the so-called all-inorganic CsPbI_3_ films processed with HI and HPbI_3_ are in fact Cs_1-*x*_DMA_*x*_PbI_3_ films, we return to the study of the latter and their behavior and performance in complete solar cells. The CsPbI_3_ film with HI addition was prepared by a conventional one-step method and using DMF as solvent^18^, actually is unintentionally-made Cs_1−*x*_DMA_*x*_PbI_3_ but we still referred it as HI-treated CsPbI_3_ in the following discussion. A small amount of HI aqueous solution was added into the CsPbI_3_ precursor after all materials were dissolved in DMF solvent. All other intentionally-made Cs_1−*x*_DMA_*x*_PbI_3_ (*x* = 0 to 0.5) films were fabricated by CsI, PbI_2_, and pre-synthesized DMAI using a solvent-engineering method^14,72^ with a mixture of DMF and DMSO as the solvent. Scanning electron microscopy (SEM) images show that the yellow-phase CsPbI_3_ film has a rough surface with big grains and pin-holes, Fig. 3a. The morphology of the neat yellow DMAPbI_3_ film is even worse, showing some long and big cracks (Fig. 3b). Fig. 3c shows the one-step method prepared CsPbI_3_ film treated with HI which clearly has much smoother surface, still containing some pin holes. The best film quality was obtained from the CsPbI_3_ film with DMA cation i.e., Cs_0.7_DMA_0.3_PbI_3_. As shown in Fig. 3d, the Cs_0.7_DMA_0.3_PbI_3_ film has a smooth surface, big grains and few pinholes. The average grain size of the Cs_0.7_DMA_0.3_PbI_3_ film is around 500 nm, which is comparable with the film thickness. This is desirable since big grains facilitate charge transfer and reduce the recombination at grain boundaries^14^. Fig. 3e shows a cross-sectional SEM of a complete device based on the Cs_0.7_DMA_0.3_PbI_3_ absorber. The 380 nm thick Cs_0.7_DMA_0.3_PbI_3_ film is sandwiched between a thin poly(3,4-ethylenedioxythiophene) polystyrene sulfonate (PEDOT:PSS) hole transporting layer and a thin fullerene (C_60_) electron transporting layer. The ultra-smooth Cs_0.7_DMA_0.3_PbI_3_ film enables the fabrication of solar cells with an inverted planar structure.

### Effects of DMA cation on solar cell performance

**Fig. 4:**
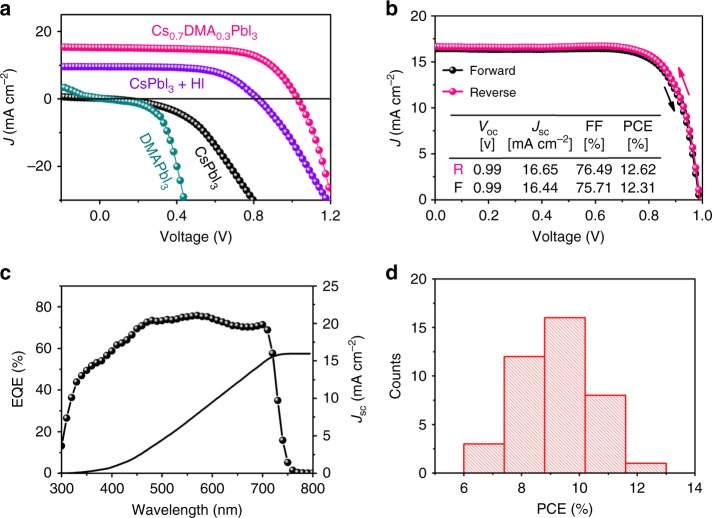
Solar cell performance. **a** *J*–*V* curves of devices using various absorbers measured under reverse voltage scans (from *V*_oc_ to 0 V). **b** *J*–*V* curves of the champion Cs_0.7_DMA_0.3_PbI_3_ solar cell measured under different voltage scan directions. **c** EQE spectrum and integrated *J*_sc_ of a Cs_0.7_DMA_0.3_PbI_3_ solar cell. **d** PCE statistics for 40 Cs_0.7_DMA_0.3_PbI_3_ solar cells

We then investigated the effects of various absorbers on device performance. Fig. 4a shows the photocurrent density-voltage (*J*–*V*) curves of the representative solar cells based on the yellow CsPbI_3_, the pale-yellow DMAPbI_3_, the black HI-treated CsPbI_3_, and the black Cs_0.7_DMA_0.3_PbI_3_ absorbers, measured under a reverse voltage scan. The performance of the solar cells based on different absorbers shows huge difference. As expected, the solar cells with yellow CsPbI_3_ and DMAPbI_3_ absorbers show very poor efficiencies. As shown in Fig. 4a, the solar cell employed the yellow CsPbI_3_ film achieved a very low PCE of 0.002% with an open-circuit voltage (*V*_oc_) of 0.05 V, a short-circuit current density (*J*_sc_) of 0.21 mA cm^−2^, a fill factor (FF) of 22.63%. A similarly low PCE of 0.001% with a *V*_oc_ of 0.03 V, a *J*_sc_ of 0.30 mA cm^−2^, and an FF of 14.23% was obtained for the solar cell employing the yellow DMAPbI_3_ film. Both CsPbI_3_ and DMAPbI_3_ devices were almost short-circuited, due to the poor film quality, non-perovskite structure, and limited light absorption due to their wide band gaps. The device performance of the so-called black CsPbI_3_ was significantly enhanced after adding HI into the perovskite precursor. The HI-treated CsPbI_3_ solar cell achieved a *V*_oc_ of 0.82 V, a *J*_sc_ of 9.56 mA cm^−2^, an FF of 61.56%, and a PCE of 4.84%, which is comparable with other reports^18,31^. The enhanced performance can be mainly attributed to the better morphology and the unintended incorporation of DMA cation which can stabilize the Cs_1−x_DMA_x_PbI_3_ perovskite as a black phase. Nevertheless, adding HI into the perovskite precursor makes it difficult to control the amount of DMA in the final film in a consistent manner, so after realizing the role of HI in the film formation the main focus was given in the easily reproducible fabrication of devices containing controlled amounts of DMA. As expected, the solar cells based on the designed Cs_0.7_DMA_0.3_PbI_3_ absorber yielded much better performance. A significantly enhanced PCE of 10.39% with a high *V*_oc_ of 1.03 V, a *J*_sc_ of 15.43 mA cm^−2^, an FF of 65.61% was achieved for an optimized device, with both the high-quality of the films and narrow band gap contributing to the decent performance of the Cs_0.7_DMA_0.3_PbI_3_ solar cells.

We find that the amount of DMA cation incorporated in the perovskite structure of CsPbI_3_ has a huge effect on the material properties. Unlike the HI addition to DMF which results in the uncontrollable generation of DMA, it is easy to fabricate Cs_1-*x*_DMA_*x*_PbI_3_ films using precise DMA ratios. First, the film morphology was affected by different amounts of DMA. As shown in Supplementary Fig. 9, Cs_0.8_DMA_0.2_PbI_3_, Cs_0.7_DMA_0.3_PbI_3_, Cs_0.6_DMA_0.4_PbI_3_, and Cs_0.5_DMA_0.5_PbI_3_ films show different morphology. All films are compact and have smooth surface with Cs_0.7_DMA_0.3_PbI_3_ film having the biggest grains. The Cs_0.6_DMA_0.4_PbI_3_, and Cs_0.5_DMA_0.5_PbI_3_ films are compact and have smaller grains. In addition, these films show similar band baps and PL peaks but different absorption intensities (Supplementary Fig. 10). In particular, the Cs_0.7_DMA_0.3_PbI_3_ film shows the strongest absorption at long wavelength range. Supplementary Fig. 11 shows that the different films also have different XRD results. The diffraction intensities of the cubic Cs_0.6_DMA_0.4_PbI_3_ and Cs_0.5_DMA_0.5_PbI_3_ films are weaker while the extra peak at 2*θ* = 11.8° indicates that DMAPbI_3_ is present as a second phase, suggesting that the substitution of DMA for Cs is limited. Cs_0.7_DMA_0.3_PbI_3_ and Cs_0.8_DMA_0.2_PbI_3_ show the exclusive formation of the black α-CsPbI_3_ phase along with the former orienting preferentially along both (100) and (110) planes and the latter exclusively along the (110) planes. From the combined data, around 30% of DMA seems to be the optimum amount to make high-quality perovskite film. This is also reflected in the device performance of the corresponding solar cells based on the CsPbI_3_ absorbers with various amounts of DMA. Supplementary Fig. 12 shows the *J*–*V* curves of the solar cells using the Cs_0.8_DMA_0.2_PbI_3_, Cs_0.7_DMA_0.3_PbI_3_, Cs_0.6_DMA_0.4_PbI_3_, and Cs_0.5_DMA_0.5_PbI_3_ absorbers. The device performance first increases and then decreases as the DMA amount increases. As expected, the solar cell derived from the best-quality Cs_0.7_DMA_0.3_PbI_3_ film achieved the highest performance (Supplementary Table 1).

Figure 4b shows the *J*–*V* curves of the best-performing Cs_0.7_DMA_0.3_PbI_3_ solar cell measured under different voltage scan directions. This solar cell achieved a *V*_oc_ of 0.99 V, a *J*_sc_ of 16.65 mA cm^−2^, an FF of 76.49%, and therefore a high PCE of 12.62% when measured under the reverse voltage scan. A similar PCE of 12.31% with a *V*_oc_ of 0.99 V, a *J*_sc_ of 16.44 mA cm^−2^, and an FF of 75.71% was achieved for the solar cell measured under the forward voltage scan, suggesting small hysteresis behavior of our devices. External quantum efficiency (EQE) measurement was taken to confirm the high *J*_sc_ obtained from the *J*–*V* curves. The EQE spectrum of the solar cell based on the Cs_0.7_DMA_0.3_PbI_3_ absorber is shown in Fig. 4c. The cell shows high average value in the whole visible wavelength range. The *J*_sc_ integrated from the EQE curve is about 15.95 mA cm^−2^, which is very close to the *J*_sc_ measured from the *J*–*V* curves. To check the reproducibility of the device performance, we then made 40 solar cells based on the Cs_0.7_DMA_0.3_PbI_3_ absorbers. The histograms of statistics PCEs for these cells are shown in Fig. 4d, ranged from 6.73 to 12.62% due to the inhomogeneous film morphology. The 40 solar cells achieved an average PCE of 9.27 ± 1.28% with a *V*_oc_ of 1.01 ± 0.03 V, a *J*_sc_ of 15.45 ± 1.80 mA cm^−2^, and an FF of 59.40 ± 4.01%.

Furthermore, we also added the DMA cation into solutions of CsPbI_2_Br and CsPbIBr_2_ perovskites. Supplementary Fig. 13a-b shows UV-vis absorption and XRD pattern of a Cs_0.7_DMA_0.3_I_2_Br film coated on a FTO/PEDOT:PSS substrate. The Cs_0.7_DMA_0.3_I_2_Br film shows an absorption onset at around 685 nm, according to a band gap of around 1.8 eV (Supplementary Fig. 13a). The Cs_0.7_DMA_0.3_I_2_Br film has good crystalline quality and shows peaks at 14.7°, 20.5°, 29.5°, and 41.6° (Supplementary Fig. 13b), which can be indexed to the (100), (110), (200), and (220) planes. Supplementary Fig. 13c shows the *J*–*V* curve of a solar cell based on the CsPbI_2_Br absorber. The Cs_0.7_DMA_0.3_I_2_Br solar cell achieved a PCE of 5.24%, a *V*_oc_ of 1.02 V, a *J*_sc_ of 12.39 mA cm^−2^, and an FF of 41.67%. The lower *J*_sc_ is consistent with the wider band gap compared to Cs_0.7_DMA_0.3_PbI_3_. The Cs_0.7_DMA_0.3_PbIBr_2_ film has a more blue-shifted absorption onset at around 620 nm, according to a wider band gap of around 2.0 eV, Supplementary Fig. 14a. The Cs_0.7_DMA_0.3_PbIBr_2_ solar cell achieved a PCE of 2.80%, a *V*_oc_ of 1.11 V, a *J*_sc_ of 8.55 mA cm^−2^, and an FF of 29.57% (Supplementary Fig. 14c). A much lower *J*_sc_ is obtained from the Cs_0.7_DMA_0.3_PbIBr_2_ cell because of the much wider band gap. Higher performance for the Cs_1-*x*_DMA_*x*_PbI_2_Br and Cs_1−*x*_DMA_*x*_PbIBr_2_ cells is anticipated after further device optimization.

## Discussion

We have demonstrated that small cation DMA can be formed in suite by the HI induced decomposition of DMF and can subsequently stabilize the black perovskite phase of CsPbI_3_. However, the films are not really the all-inorganic CsPbI_3_ phase but the Cs_1-*x*_DMA_*x*_PbI_3_ (*x* = 0.2 to 0.5) cubic perovskite solid solution. Cs_0.7_DMA_0.3_PbI_3_ has a band gap of around 1.7 eV and can enable high efficiency solar cells. In the context of this work, we realize that DMA is likely present in many other devices reported in the past, since it can be inadvertently created from the degradation of the commonly used DMF solvent and then incorporated to some extend in the perovskite structures as an A cation. This DMF decomposition process is accelerated by acidic hydrolysis, and must be taken into account when considering the role of the solvent degradation in device performance. DMF hydrolysis therefore explains the effects of the widely used HI addition and HPbI_3_ methods to stabilize the purported all-inorganic black CsPbI_3_ perovskite. Based on above results, we show that HPbI_3_ does not exist while at the same time it is actual in situ formation of the DMA cation, which should widely exist in the perovskites processed with DMF, HI, and HPbI_3_. We anticipate that the DMA cation, which now has been revealed as a viable A-site cation, may be unsuspectingly occurring not only in "CsPbI_3_ films" but also in films of other perovskite families, for example, pure MA, MA/FA, Cs/FA, and Cs/MA/FA mixed-cation perovskites. We suggest that NMR spectroscopy be used for such films by dissolving them in pure DMF or DMSO to check for the presence of the DMA cation.

## Methods

### Device fabrication

For the DMAI synthesis, DMF was slowly added into the HI solution with continue stirring at 0 °C for 30 min. Water and excess DMF in solution was removed by low-vacuum rotary evaporation and then a white polycrystalline powder (DMAI powder) was obtained. Pre-cleaned FTO substrates were treated with UV-Ozone for 30 min. PEDOT:PSS films were coated on the FTO substrates with a spin-rate of 4000 rpm for 30 s and then annealing for 30 min at 150 °C. Perovskite films were deposited on FTO/PEDOT:PSS substrates in a N_2_-filled glovebox. Neat CsPbI_3_, neat DMAPbI_3_, and Cs_1−x_DMA_x_PbX_3_ (*X* = I, Br) films were prepared by a solvent-engineering method^14^ at a spin rate of 4000 rpm for 60 s. 0.7 ml of diethyl ether was quickly dropped onto the rotating substrates during the spin-coating process. CsPbI_3_ films with HI addition were prepared by a one-step method at a spin rate of 2000 rpm for 60 s^18^. The neat CsPbI_3_ precursor was prepared by dissolving 260 mg of CsI (99.999%, Sigma-Aldrich) and 461 mg of PbI_2_ (beads, 99.999%, Sigma-Aldrich) in 0.8 ml of DMF (anhydrous, 99.8%, Sigma-Aldrich) and 0.2 ml of DMSO (anhydrous,≥ 99.9%, Sigma-Aldrich). The neat DMAPbI_3_ precursor was prepared by dissolving 173 mg of DMAI and 461 mg of PbI_2_ in 0.8 ml of DMF and 0.2 ml of DMSO. The Cs_1−*x*_DMA_*x*_PbI_3_ precursor was prepared by dissolving (1−*x*) × 260 mg of CsI, *x* × 173 mg of DMAI, and 461 mg of PbI_2_ in 0.8 ml of DMF and 0.2 ml of DMSO. The CsPbI_3_ precursor with HI addition was prepared by dissolving 260 mg of CsI and 461 mg of PbI_2_ in 2 ml of DMF and then adding 3.3 vol% HI (57 wt.% in H_2_O, 99.95%, Sigma-Aldrich) into the dissolved solution. All the films were annealed for 2 min at 60 °C and then 5 min at 100 °C on a hot plate. To complete the devices, a thin C_60_ (20 nm), a thin 2,9-dimethyl-4,7-diphenyl-1,10-phenanthroline (BCP) (5 nm), and a Ag (80 nm) films were sequentially thermal evaporated on top of the absorber layers using a metal mask. The active area of the solar cells was 0.09 cm^2^.

### Film and device characterization

^1^H-NMR spectra were measured with Bruker Avance III 600 MHz system with BBI probe. The morphology of the films and devices was examined on a high-resolution field emission SEM (Hitachi SU8030). XRD patterns of the films were characterized by a Rigaku Miniflex600 pXRD (Cu Kα graphite, *λ* = 1.5406 Å) operating at 40 kV/15 mA with a Kβ foil filter. PL spectra were taken on a Horiba LabRAM HR Evolution confocal Raman microscope spectrometer (600 g mm^−1^ diffraction grating) equipped with a diode continuouswave laser (473 nm, 25 mW) and a Synapse charge-coupled device camera. UV-vis absorption spectra of the films were measured with a Shimadzu UV-3600 UV-vis NIR spectrometer operating in the 200–2000 nm region at room temperature. EQE spectrum was obtained on an Oriel model QE-PV-SI instrument equipped with a NIST-certified Si diode. *J*–*V* curves were recorded using a Keithley model 2400 instrument under AM1.5 G simulated irradiation with a standard solar simulator (Abet Technologies). *J*–*V* curves were measured from 1.5 V to −0.2 V (reverse) or from −0.2 V to 1.5 V (forward) with an integration time of 16.67 ms and a voltage step of 11.4 mV. The light intensity of the solar simulator was calibrated by a NREL-certified monocrystalline silicon solar cell.

## Electronic supplementary material

Supplementary Information

## Data Availability

The authors declare that the main data supporting the findings of this study are available within the article and its Supplementary Information. Extra data are available from the authors upon request.
