# Supplementary material for: Myths and reality of HPbI3 in halide perovskite solar cells
Source: Nat Commun. 2018 Nov 14;9:4785. doi: 10.1038/s41467-018-07204-y (PMC6235929; doi:10.1038/s41467-018-07204-y)
Supplement: Supplementary file 1 — Supplementary Information [file 41467_2018_7204_MOESM1_ESM.docx]

**Supplementary Information**

**Myths and reality of HPbI_3_ in halide perovskite solar cells**

Weijun Ke,^†^ Ioannis Spanopoulos,^†^ Constantinos C. Stoumpos,^†^* and Mercouri G. Kanatzidis^†^*

^†^Department of Chemistry, Northwestern University, Evanston, IL 60208, United States

E-mail: konstantinnos.stoumpos@northwestern.edu; m-kanatzidis@northwestern.edu


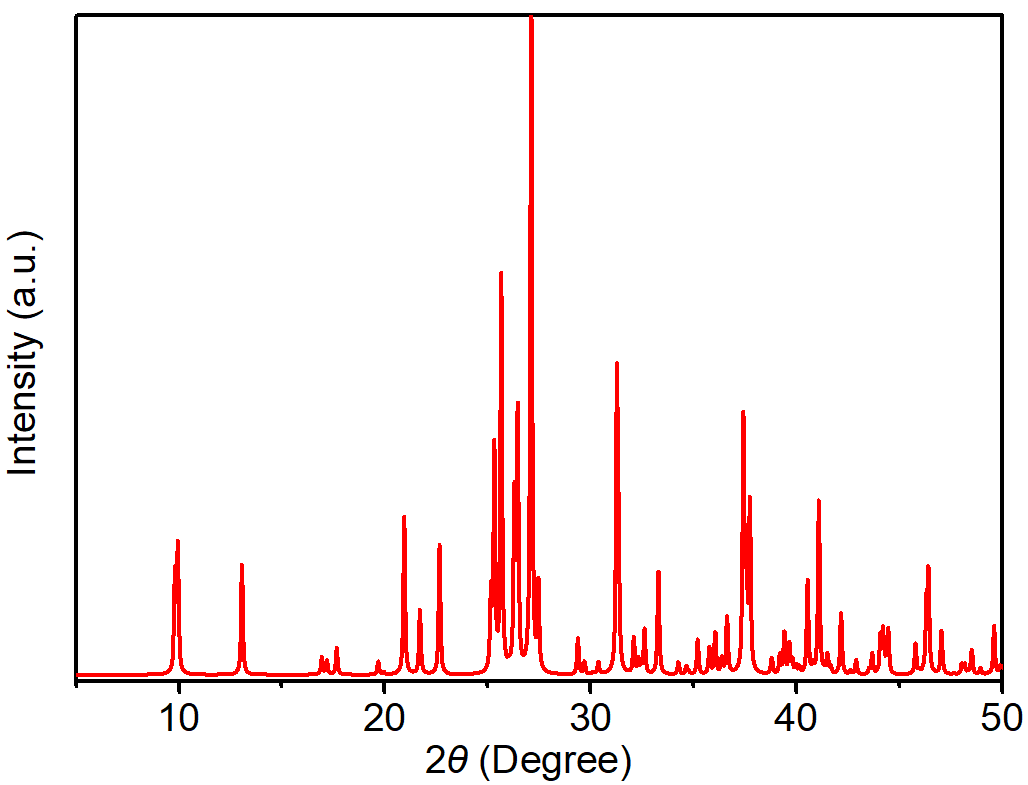


Supplementary Figure 1. Simulated XRD pattern of yellow δ-CsPbI_3_ (a=10.4594(2) Å b=4.8022(0) Å c=17.7778(2) Å)


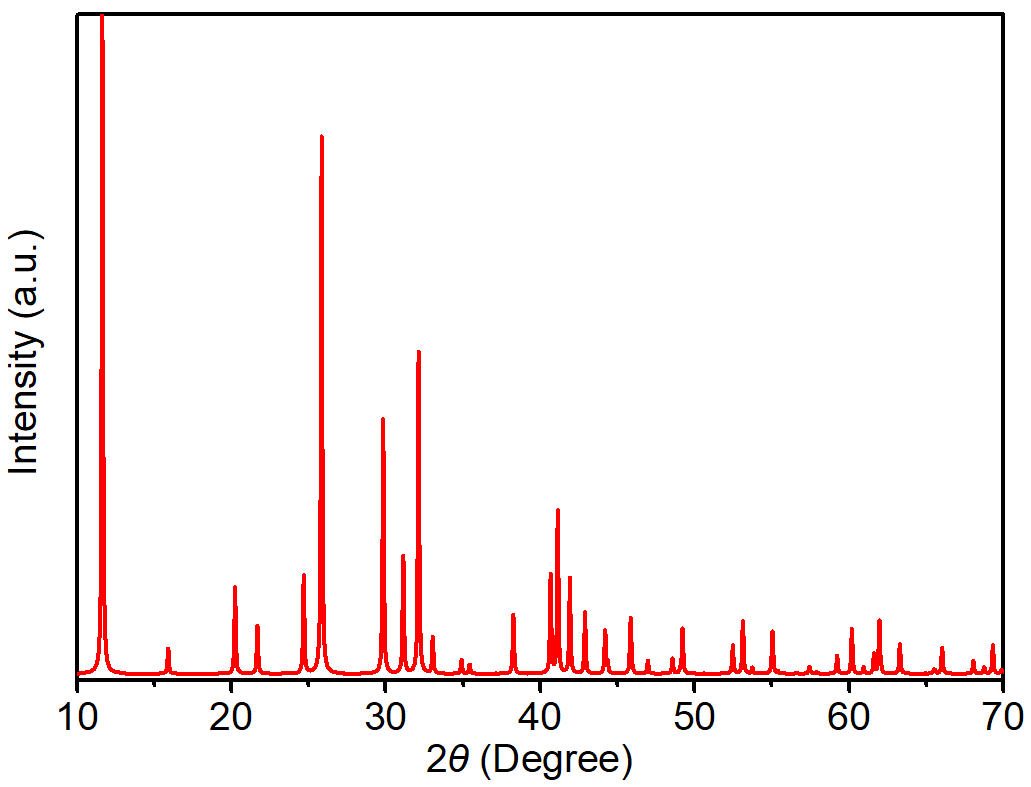


Supplementary Figure 2. Simulated XRD pattern of yellow γ-DMAPbI_3_.


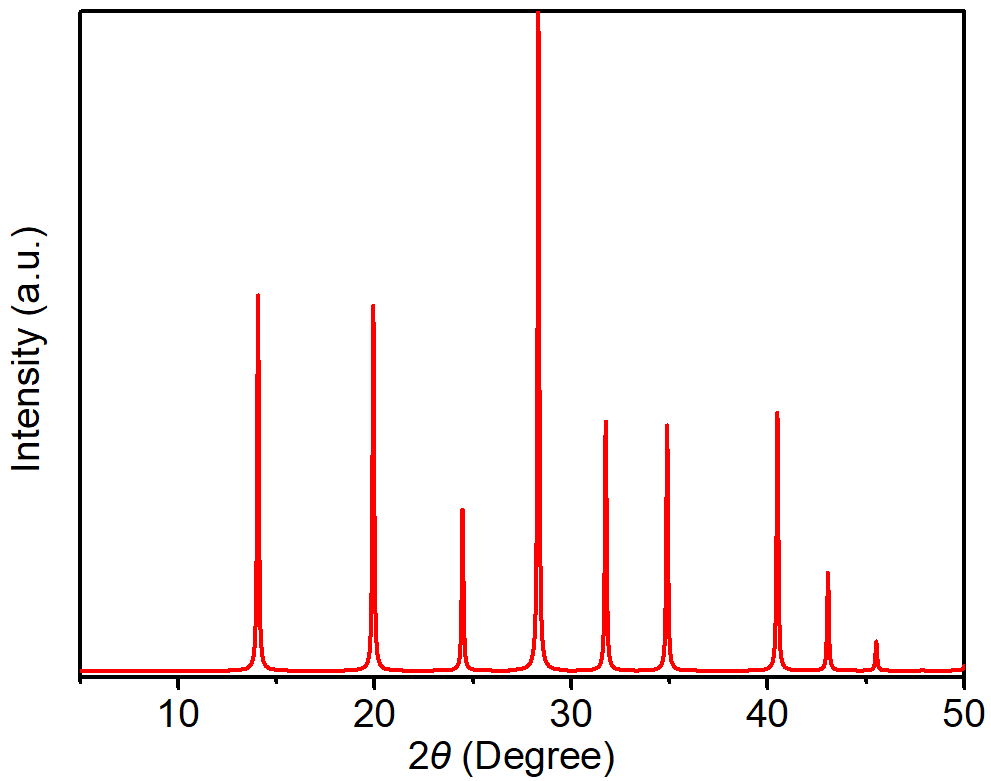


Supplementary Figure 3. Simulated XRD pattern of black cubic α-CsPbI_3_ at 645°C (a=b=c=6.2966(0) Å).


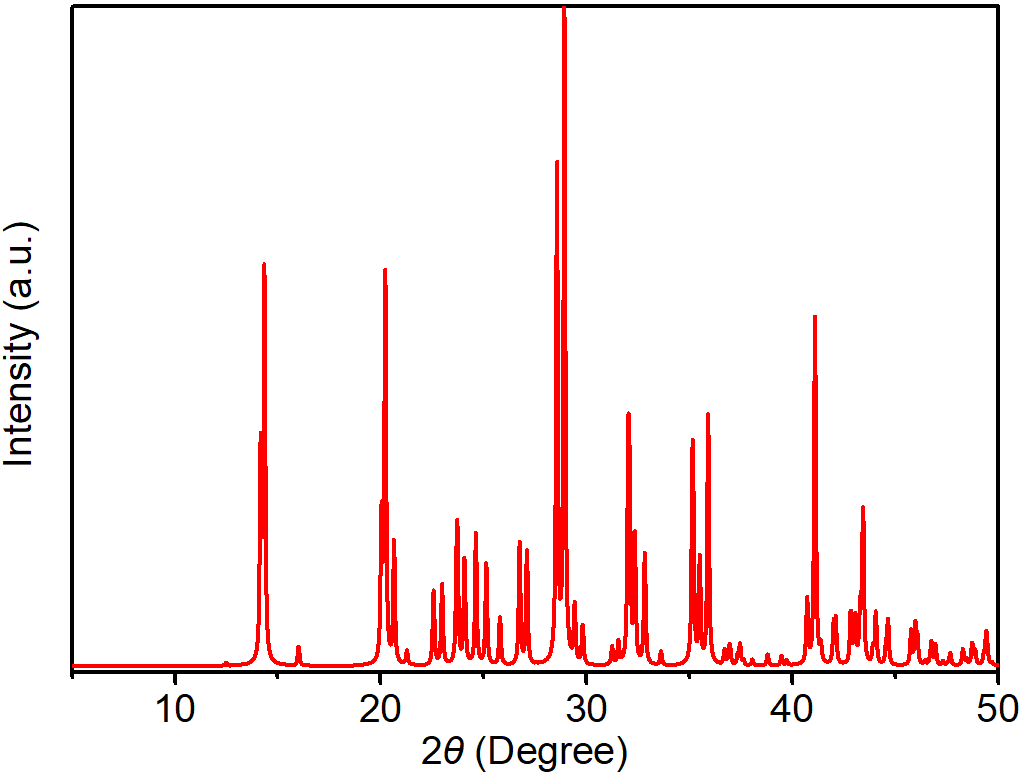


Supplementary Figure 4. Simulated XRD pattern of black γ-CsPbI_3_ (a=8.6198(1) Å b=8.8518(1) Å c=12.5013(1) Å).


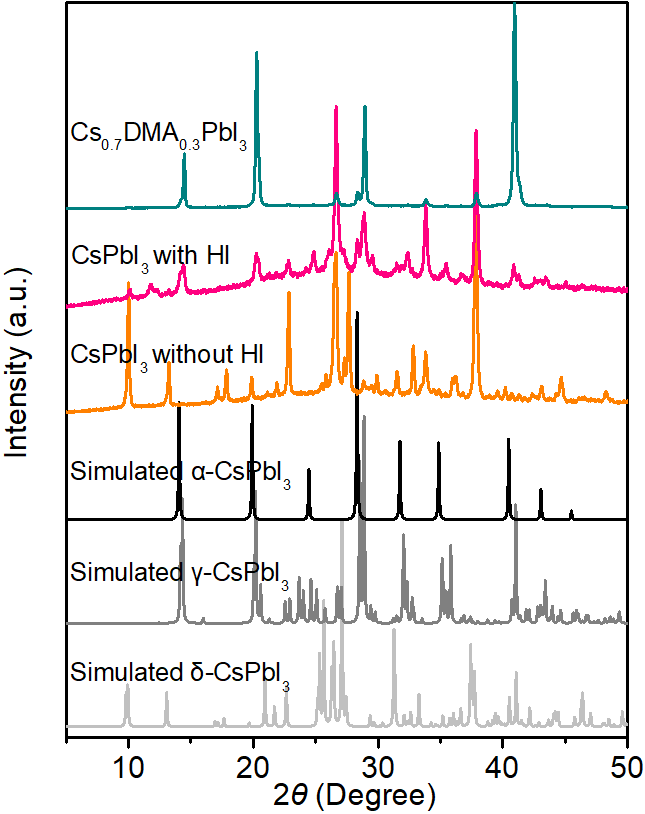


Supplementary Figure 5. Comparison of the simulated and measured PXRD patterns of CsPbI_3_ with different phases. The perovskite phase form only when DMA is present.


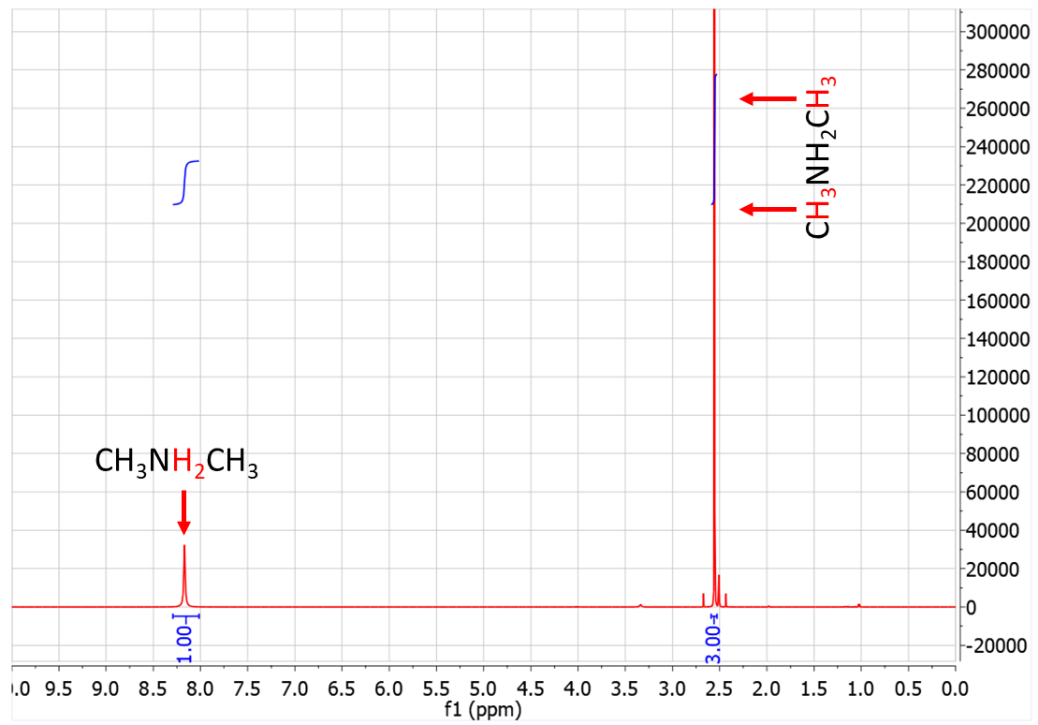


Supplementary Figure 6. NMR spectrum of DMAI (98%, Sigma-Aldrich) polycrystalline powder dissolved in DMSO-d_6_.


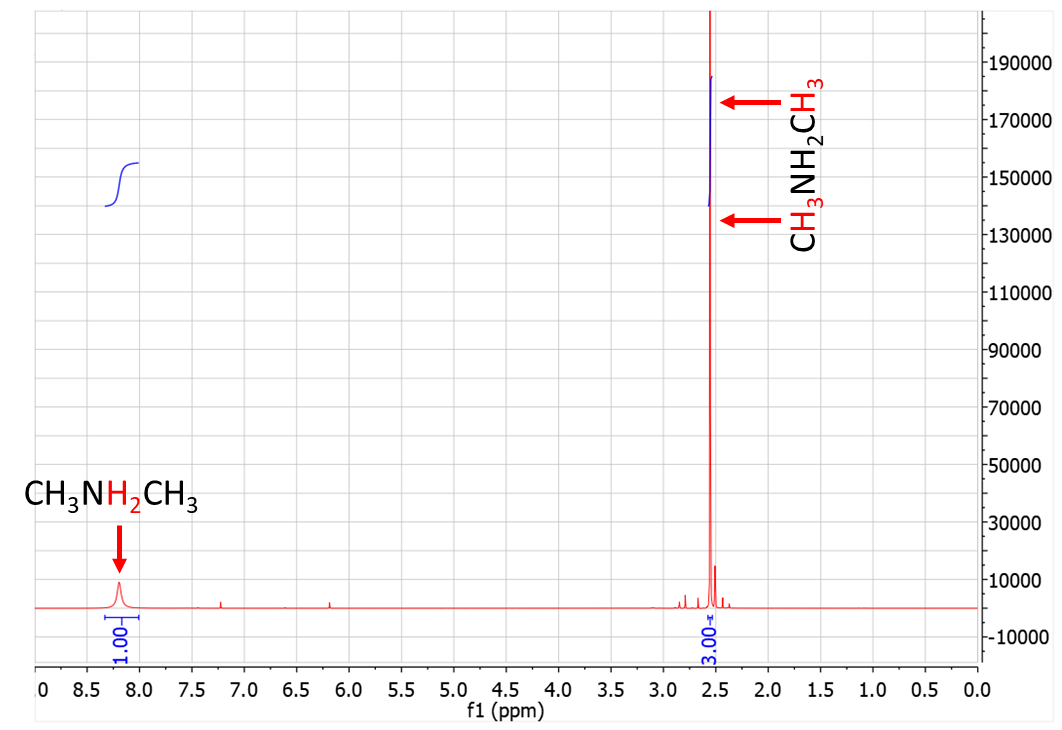


Supplementary Figure 7. NMR spectrum of the powder obtained from scratching away HI-treated CsPbI_3_ films, which was dissolved in DMSO-d_6_.


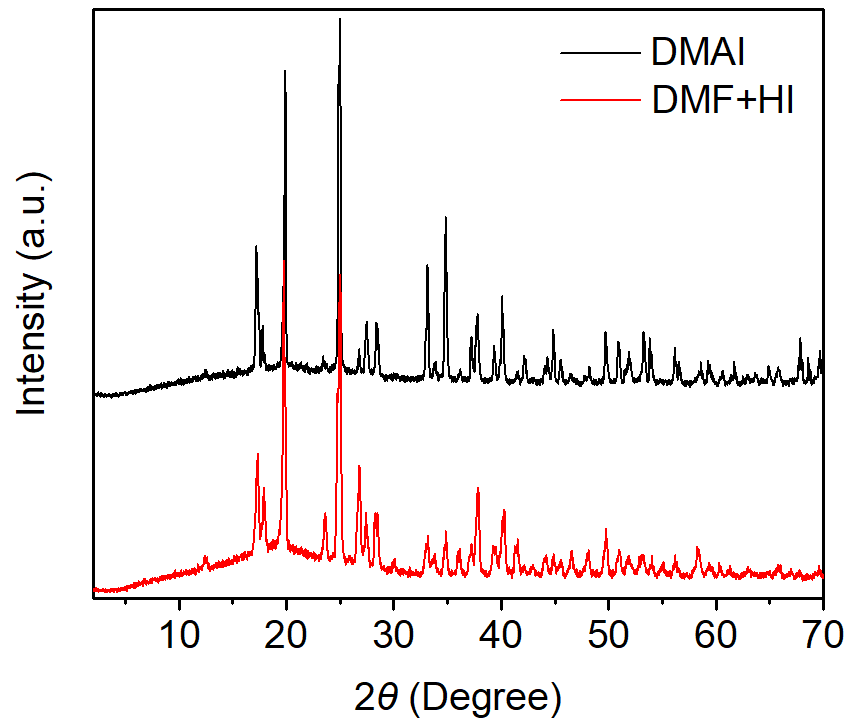


Supplementary Figure 8. XRD patterns of DMAI (98%, Sigma-Aldrich) and the compound synthesized from the reaction of DMF and HI.


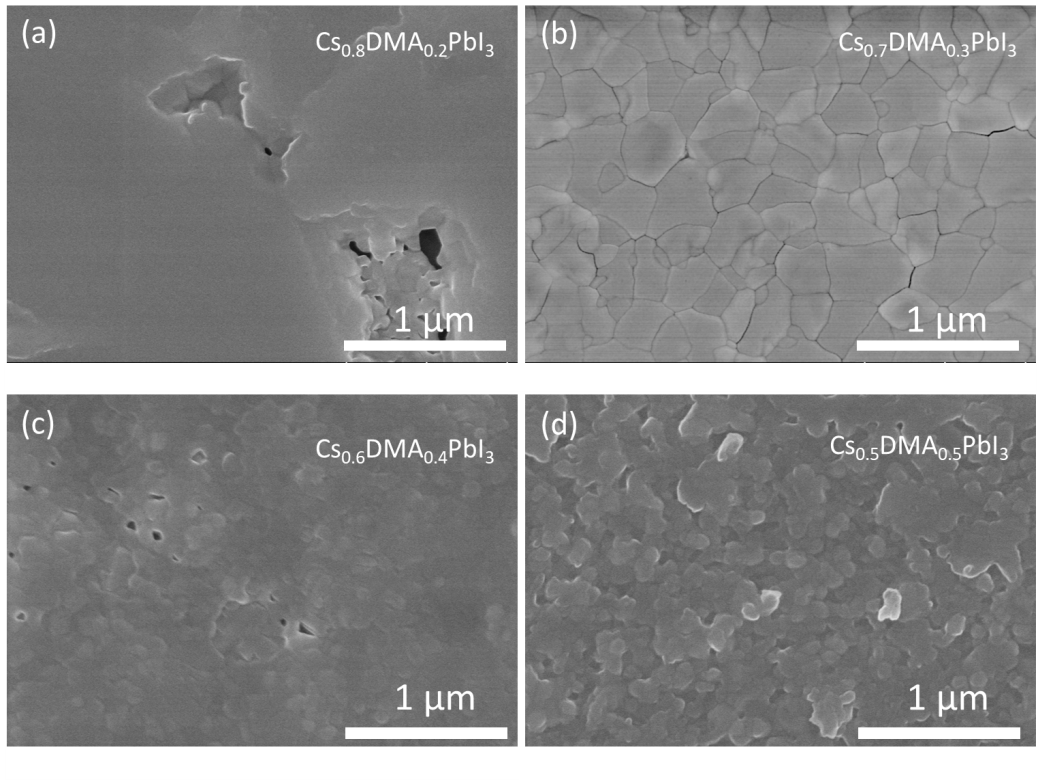


Supplementary Figure 9. Top view SEM images of (a) a Cs_0.8_DMA_0.2_PbI_3_, (b) a Cs_0.7_DMA_0.3_PbI_3_, (c) a Cs_0.6_DMA_0.4_PbI_3_, and (d) a Cs_0.5_DMA_0.5_PbI_3_ films deposited on FTO/PEDOT:PSS substrates.


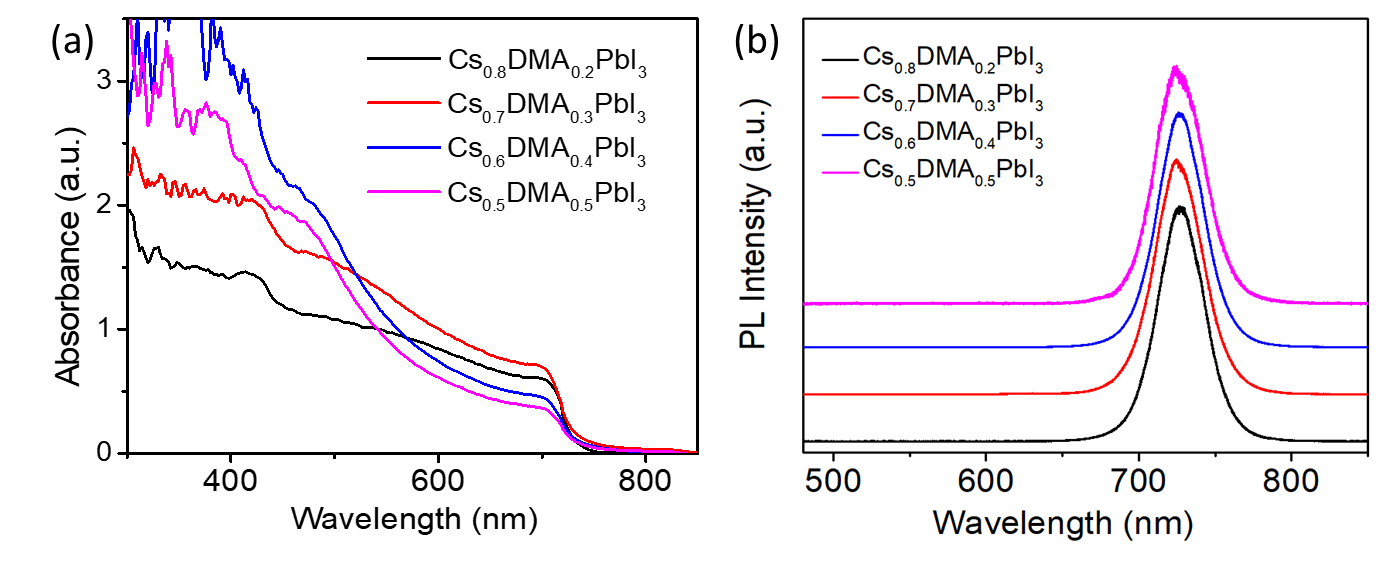


Supplementary Figure 10. (a) UV-vis absorption and (b) PL spectra of a Cs_0.8_DMA_0.2_PbI_3_, a Cs_0.7_DMA_0.3_PbI_3_, a Cs_0.6_DMA_0.4_PbI_3_, and a Cs_0.5_DMA_0.5_PbI_3_ films deposited on FTO/PEDOT substrates.


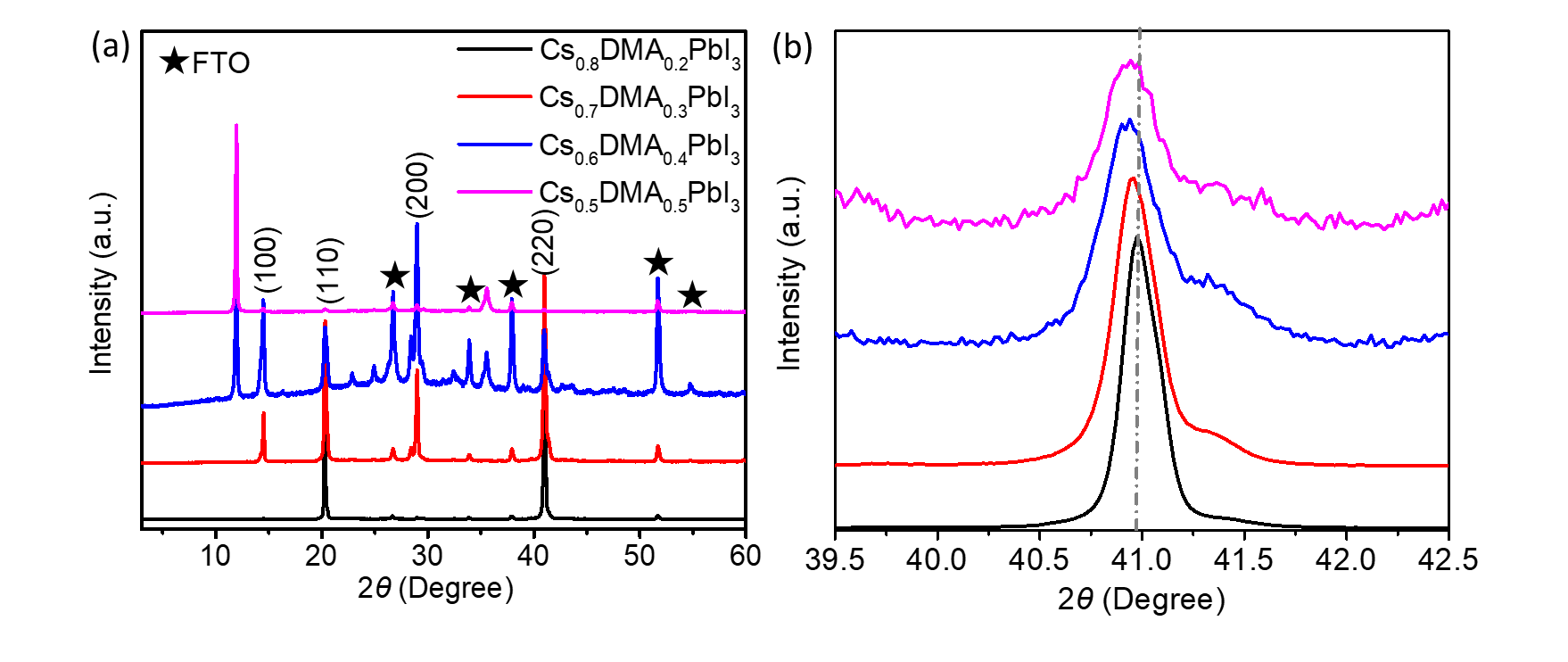


Supplementary Figure 11. (a, b) XRD patterns of a Cs_0.8_DMA_0.2_PbI_3_, a Cs_0.7_DMA_0.3_PbI_3_, a Cs_0.6_DMA_0.4_PbI_3_, and a Cs_0.5_DMA_0.5_PbI_3_ films deposited on FTO/PEDOT substrates.


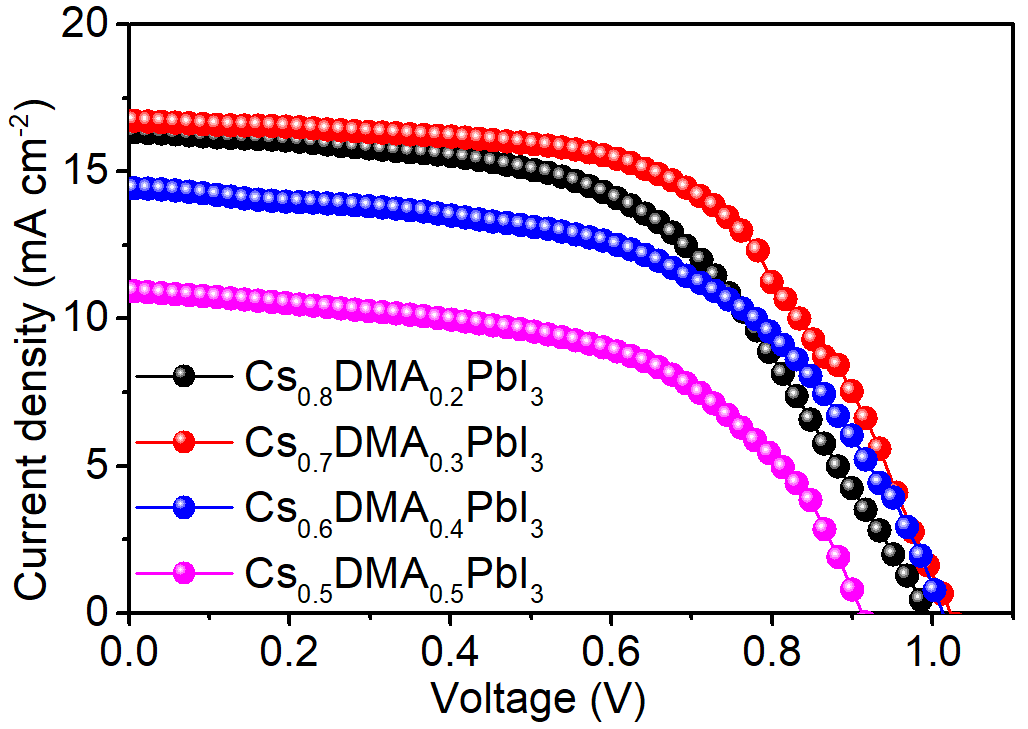


Supplementary Figure 12. *J*-*V* curves of (a) a Cs_0.8_DMA_0.2_PbI_3_, (b) a Cs_0.7_DMA_0.3_PbI_3_, (c) a Cs_0.6_DMA_0.4_PbI_3_, and (d) a Cs_0.5_DMA_0.5_PbI_3_ solar cells measured under reverse voltage scans.


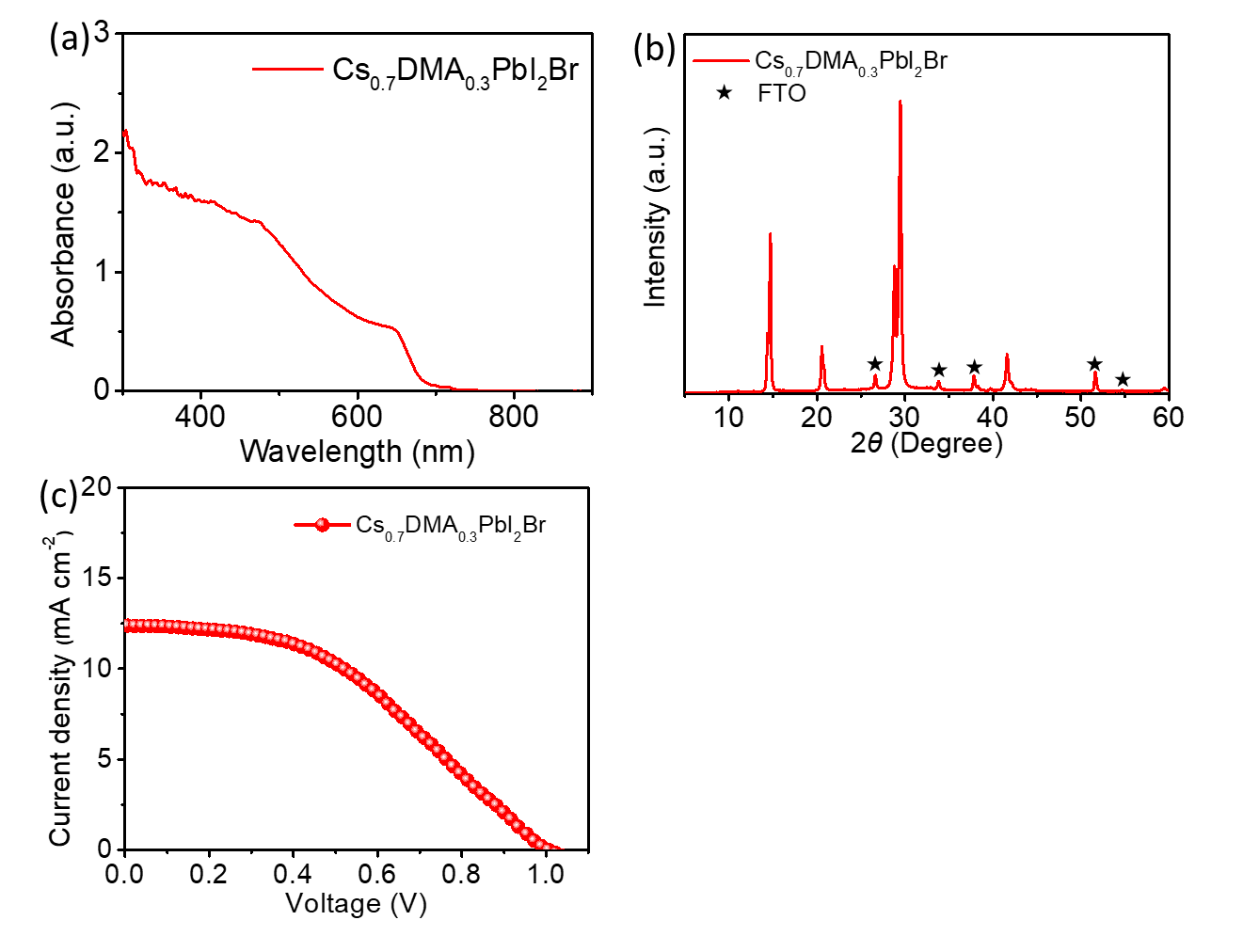


Supplementary Figure 13. (a) UV-vis absorption spectrum and (b) XRD pattern of a Cs_0.7_DMA_0.3_PbI_2_Br film deposited on an FTO/PEDOT substrate. (c) *J*-*V* curve of a Cs_0.7_DMA_0.3_PbI_2_Br-based solar cell measured under a reverse voltage scan.


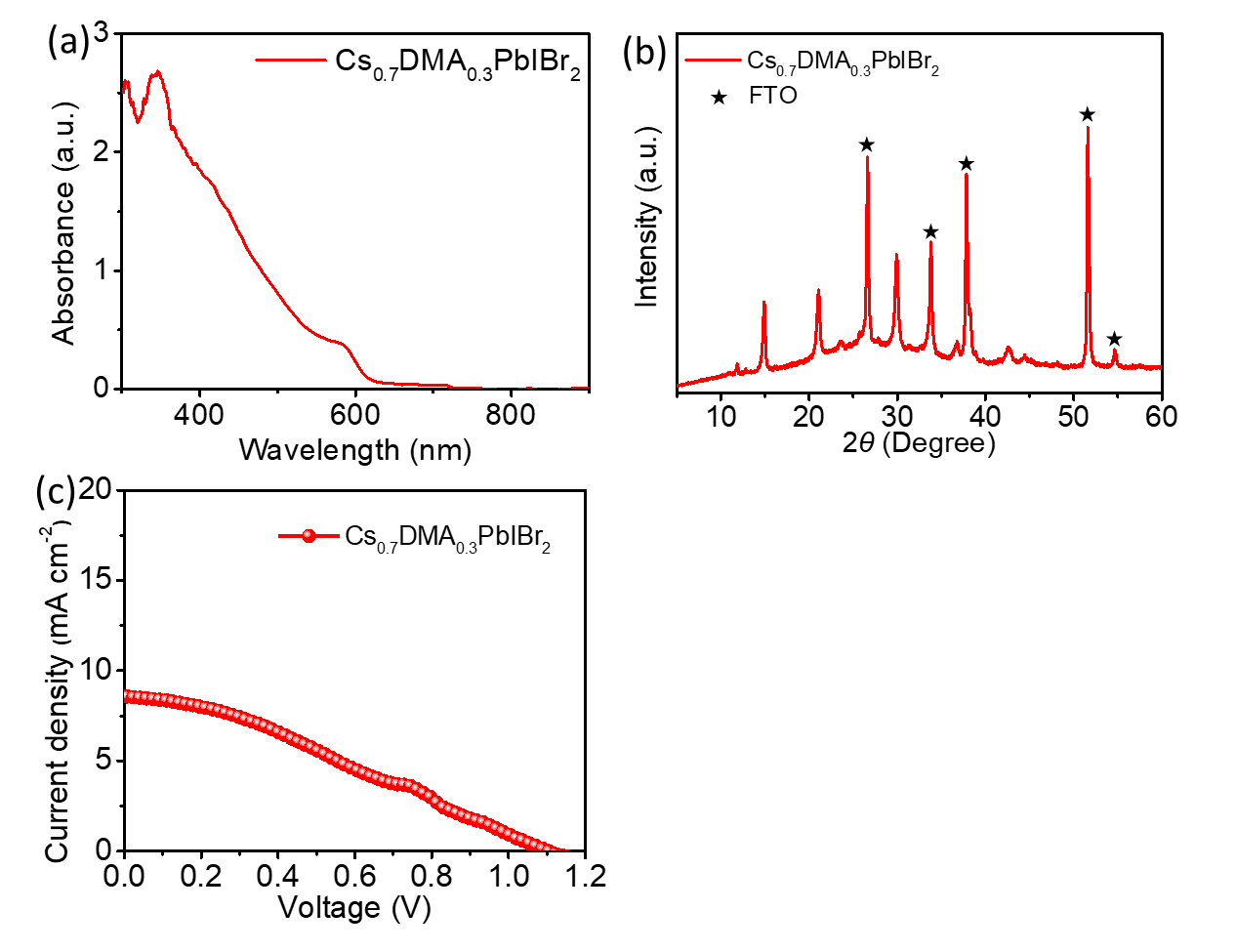


Supplementary Figure 14. (a) UV-vis absorption spectrum and (b) XRD pattern of a Cs_0.7_DMA_0.3_PbIBr_2_ film deposited on an FTO/PEDOT substrate. (c) *J*-*V* curve of a Cs_0.7_DMA_0.3_PbIBr_2_-based solar cell measured under a reverse voltage scan.

Supplementary Table 1. Summary of the photovoltaic parameters of the CsPbI_3_ solar cells with various amounts of DMA.

|  | *V*_oc_ | *J*_sc_ | FF | PCE |
| --- | --- | --- | --- | --- |
|  | [V] | [mA cm^-2^] | [%] | [%] |
| Cs_0.8_DMA_0.2_PbI_3_ | 0.99 | 16.34 | 53.92 | 8.75 |
| Cs_0.7_DMA_0.3_PbI_3_ | 1.02 | 16.72 | 59.00 | 10.08 |
| Cs_0.6_DMA_0.4_PbI_3_ | 1.01 | 15.97 | 56.09 | 9.06 |
| Cs_0.5_DMA_0.5_PbI_3_ | 0.91 | 10.96 | 55.06 | 5.50 |
